# Utilizing plasma exchange for severe cytokine release syndrome after CAR-T cell therapy: clinical experience and literature insights

**DOI:** 10.3389/fimmu.2025.1597512

**Published:** 2025-06-24

**Authors:** Jiasi Zhang, Dali Xie, Meiyan Tang, Qiang Gong, Xingqin Huang, Ju Li, Shuangnian Xu, Nan Luo, Huiling Sun, Fangfang Tan

**Affiliations:** ^1^ Center of Haematology, Southwest Hospital, Army Medical University, Chongqing, China; ^2^ Department of General Surgery, PLA Middle Military Command General Hospital, Wuhan, China

**Keywords:** CAR-T, CRS, plasma exchange, cytokines, hematological malignancies

## Abstract

Cytokine release syndrome (CRS) is a severe complication following Chimeric Antigen Receptor T-cell (CAR-T) therapy, characterized by an excessive inflammatory response triggered by the activation of CAR-T cells. Clinically, approaches like tocilizumab and corticosteroids are commonly used to treat CRS. However, those methods might be insufficient, particularly in treating severe CRS patients (grade 3-4). Nowadays, therapeutic plasma exchange (PE) has been used as a promising adjunctive therapy to treat severe CRS, as it can rapidly remove circulating inflammatory cytokines and immune complexes which contribute to CRS progression. To summarize the characteristics and clinical usage of PE, we provide the experiences of 3 PE cases from our institution and 19 PE cases from relevant literature. In this review, we concluded that PE is effective in reducing elevated serum cytokine levels and alleviating CRS symptoms such as fever, hypotension, and neurotoxicity. Furthermore, we discuss the principles and development of PE and compare CAR-T-induced CRS with CRS caused by viral infections. In addition, PE demonstrates clear advantages over other blood purification techniques including hemofiltration (HF) and hemodiafiltration (HDF), particularly in its ability to remove large-molecular cytokines and immune complexes. To conclude, PE presents a promising therapeutic approach for managing severe CRS after CAR-T therapy, especially when standard treatments have failed.

## Introduction

Chimeric Antigen Receptor T-cell (CAR-T) therapy has shown great efficacy in treating hematologic malignancies, such as B-cell acute lymphoblastic leukemia (B-ALL), Non-Hodgkin Lymphoma (NHL) and Multiple Myeloma (MM). CAR-T involves the genetic modification of T-cells to express a receptor targeting specific tumor antigens, such as CD19 or BCMA (1–5). Despite its impressive clinical efficacy, CAR-T cell therapy is still accompanied with significant adverse effects, which might restrict its large-scale clinical applications (6, 7). Among those adverse events, CRS is the most common one, which is characterized by the systemic inflammatory responses caused by the rapid activation and proliferation of CAR-T cells after interacting with cancer cells. The severity of CRS varies among patients and is associated with tumor burden and CAR-T cell proliferation kinetics (8).

CRS progresses through several stages, starting with mild symptoms like fever and fatigue. As it worsens, patients may develop hypotension, hypoxia and multi-organ dysfunction. In severe cases, neurological symptoms such as confusion and seizures may occur. CRS is classified into different grades, with grade 1 being mild and grade 4 being life-threatening.

The standard management of CRS generally involves tocilizumab (an IL-6 receptor antagonist) and corticosteroids, which aim to alleviate the inflammatory response (9–11). However, except for IL-6, a large number of other cytokines play critical roles in the progress of CRS, such as IL-1, IL-2, IL-10, TNF-α and IFN-γ (12, 13). The conventional therapy like tocilizumab cannot remove those pre-existing cytokines, might lead to treatment resistance in 20-30% severe CRS patients (14). For example, persistently elevated IL-10 and TNF-α levels post-tocilizumab were reported to correlate with refractory hypotension and neurotoxicity (15). Besides, the corticosteroids can only suppress the inflammatory response but cannot eliminate those existing cytokines. This might lead to the fact that a subset of patients with severe CRS does not respond adequately to these first-line therapies, underscoring the need for alternative strategies to directly eliminate those inflammatory cytokines.

As an important blood purification technique, PE has emerged as a potential adjunctive approach to treat CRS (16–18). By replacing patient plasma with albumin or fresh frozen plasma, PE can rapidly remove cytokines such as IL-6 and TNF-α and alleviate the organs damage caused by those cytokines. Previous reports demonstrate that PE can effectively reduce the IL-6 levels within 24 hours and resolve clinical symptoms in about 80% CRS patients (16, 17).

While PE has shown promise in the management of CRS after CAR-T therapy, its clinical application remains under-researched. This review integrates our institutional experience with 3 severe CRS cases and a systematic analysis of 19 published cases to demonstrate the therapeutic role of PE in treating CAR-T associated CRS. Additionally, the review concludes with the differences between PE and other blood purification techniques, and discusses the timing, replaced plasma volume and future directions in PE for CAR-T-related CRS.

## Clinical experience

From 2017, patients with relapsed/refractory acute lymphoblastic leukemia were enrolled in the clinical trial treating with anti-CD19 CAR-T cell (NCT02349698) (18, 19). This study was approved by the institutional review board (IRB) of the Southwest Hospital of Army Medical University, and all enrolled patients signed informed consents. During this study, all patients experienced different degrees of CRS, and most of them successfully recovered from symptomatic treatments such as tocilizumab and corticosteroids. However, there were 3 patients experiencing severe CRS (grade 3-4) and failing after tocilizumab and corticosteroids. Eventually, PE was administered, and CRS symptoms were rapidly relieved.

These 3 patients were all diagnosed with r/r ALL and experienced grade 3–4 cytokine release syndrome (CRS) following CD19 CAR-T cell therapy. Although those patients were administrated with standard treatments, including tocilizumab and corticosteroids, their symptoms like fever and hypotension persisted. Besides, the levels of cytokines remained significantly elevated. As a result, all 3 patients underwent the treatment of PE, while the number of PE sessions and the volume of plasma exchanged varied. As shown in **Table 1**, patient 1 (P1) underwent two sessions of PE on days 6 and 7 post-infusion, with a total PE volume of 1,800 ml; patient 2 (P2) and patient 3 (P3) received one session of PE on days 8 and 7 respectively, exchanging 750 ml and 600 ml of plasma.

**Table 1:** The basic characteristics and clinical outcomes of 3 ALL patients treating with PE.

Patient	Gender	Age (years)	CRS grade at the time point of PE	Neurotoxicity grade before PE	Neurotoxicity grade after PE	IL-6 levels before PE (pg/ml)	Amount increase of IL-6 before PE (pg/ml)	Times of PE	Day of PE after infusion	Volume of PE (ml)	Outcome
P1	Male	18	4	2	1	> 10000	> 2000	2	6	1800	Recovery
P2	Male	16	3	1	0	> 5000	> 2000	1	8	750	Recovery
P3	Female	8	4	2	0	2523	> 2000	1	7	600	Recovery

**Figure 1:**
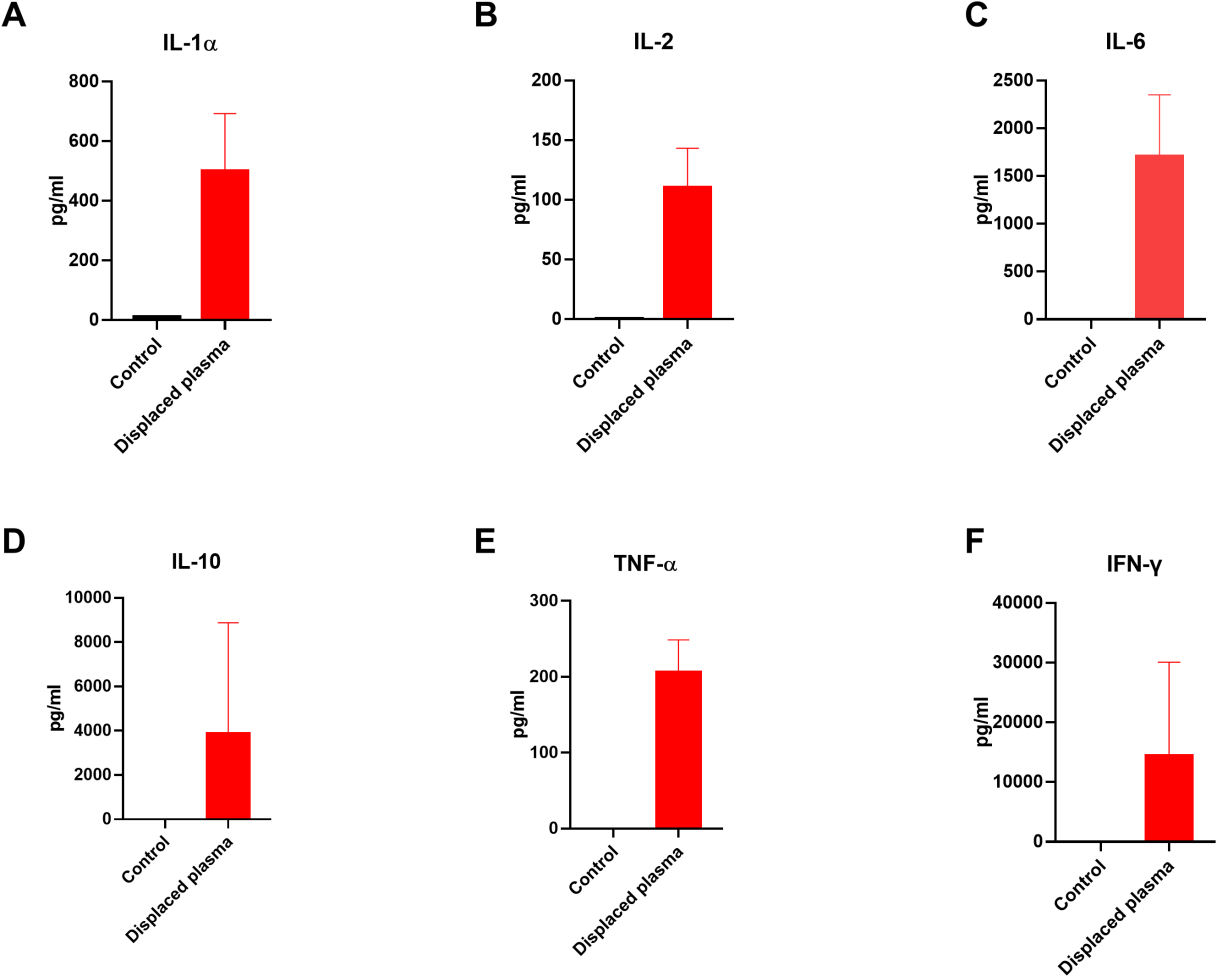
Levels of cytokines in the fresh plasma and displaced plasma before and after PE in 3 patients. **(A-F)** The concentrations of IL-1α, IL-2, IL-6, IL-10, TNF-α and IFN-γ in the plasma. We only provide the descriptive analysis instead of statistical analysis about this difference, due to the small sample size. ELISA was used to detect the concentration of these cytokines in the plasma.

**Figure 2:**
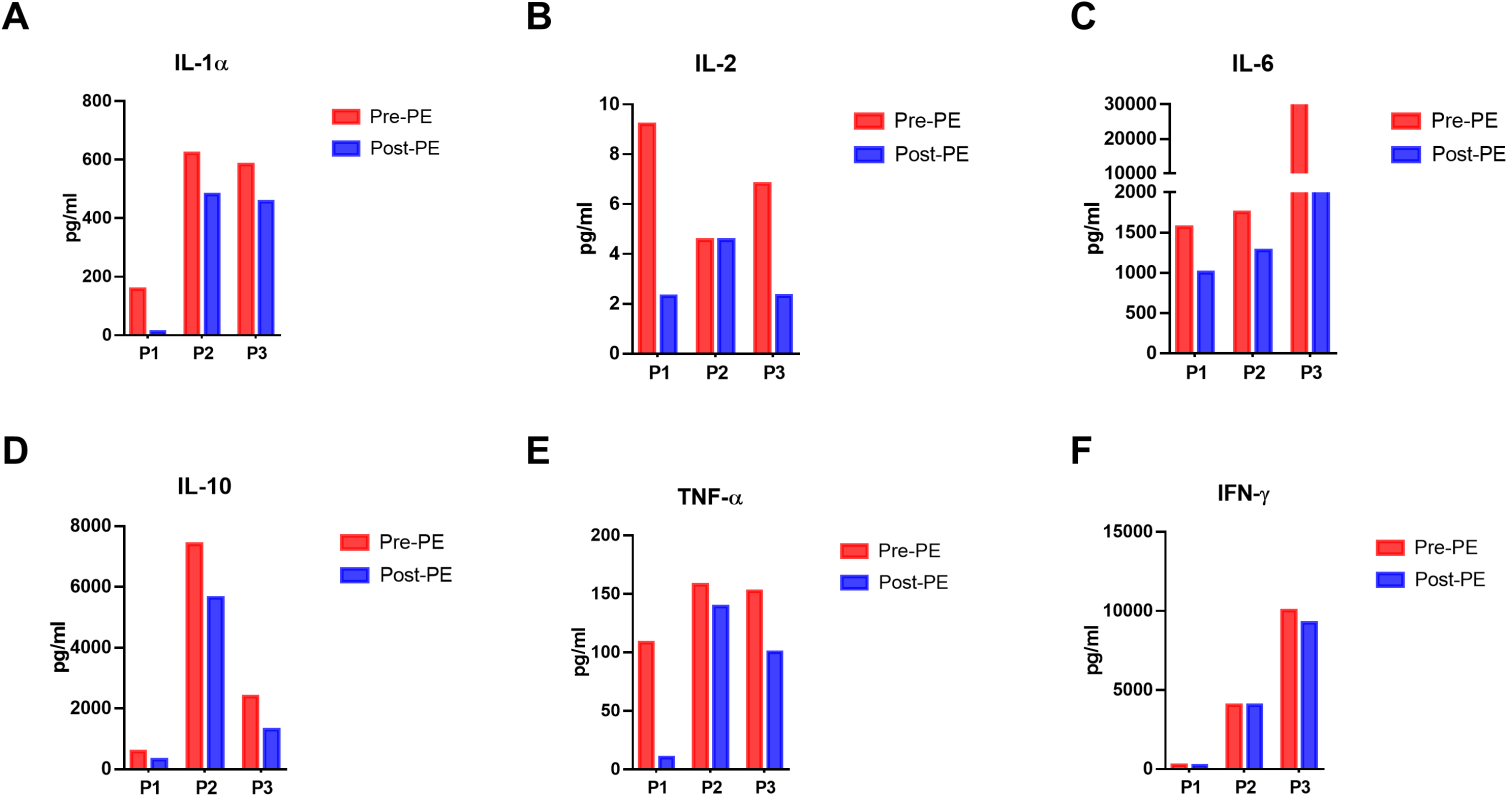
Dynamics of cytokines in the peripheral blood of patients before and immediately after PE in 3 patients. **(A-F)** The dynamics of IL-1α, IL-2, IL-6, IL-10, TNF-α and IFN-γ in the peripheral plasma before and after PE. ELISA was used to detect the concentration of these cytokines in the peripheral blood.

During the process of PE, we collected the sample of fresh and displaced plasma before and after the PE and examined the cytokine levels in the plasma. Enzyme-Linked Immunosorbent Assay (ELISA) is used to detect and quantify cytokines in biological samples. As shown in **Figure 1**, the amounts of inflammatory cytokines like IL-1α, IL-2, IL-6, IL-10, TNF-α and IFN-γ were higher than those in the fresh plasma, indicating the direct effectiveness of PE to remove those cytokines. Besides, we monitored the changes in cytokine concentrations in the plasma of the patients post-PE (as shown in **Figure 2**). The results revealed that PE led to a reduction in IL-6 and other inflammatory cytokines. This reduction was accompanied by a marked improvement in clinical symptoms, such as fever and hypotension. Importantly, the grade of neurotoxicity was obviously relieved in these 3 patients after the PE treatment, which might be explained by the reduction of the cytokines burden and protection of the blood-brain barrier from the consistent damage of those cytokines (20). However, we only provide the descriptive analysis instead of statistical analysis about this difference, due to the small sample size.

Overall, all 3 patients recovered fully, with no long-term complications, demonstrating the potential of PE as an effective adjunctive therapy in managing severe CRS following CAR-T cell therapy. This case series suggested that PE could provide rapid relief in cases of CRS and might be considered for use in combination with standard CRS management strategies especially when severe CRS occurred.

## Literature review

This literature review summarizes the use of therapeutic PE in patients experiencing severe CRS after CAR-T cell therapy, a complication triggered by the excessive inflammatory response following CAR-T cell activation (16, 17, 21). Accordingly, 19 patients with severe CRS had been treated with PE after CAR-T cell therapy. Most of them in these studies received CAR-T products targeting CD19, primarily for the treatment of B-ALL. The details of those patients were summarized in **Table 2**.

**Table 2:** Summary of cases of PE treating CRS after CAR-T cells infusion.

Patients	Diagnosis	Target of CAR-T	CRS grade before PE	Neurotoxicity grade after PE	Times of PE	Day of PE after infusion	Tocilizumab before PE	Corticosteroid before PE	Change of IL-6 after PE	Outcome	Authors
P1	AML	CLL1	2	1	4	6	YES	YES	Decrease	Recovery	Yedi Pu et al
P2	AML	CLL1	3	1	3	5	YES	YES	Decrease	Recovery	Yedi Pu et al
P3	AML	CLL1	4	2	3	8	YES	YES	Increase	DEATH	Yedi Pu et al
P4	ALL	CD19	4	1	2	14	YES	YES	Decrease	Recovery	Yedi Pu et al
P5	ALL	CD19	4	0	2	7	YES	YES	Decrease	Recovery	Yedi Pu et al
P6	ALL	CD19	4	3	1	15	YES	YES	Increase	DEATH	Yedi Pu et al
P7	ALL	CD19	4	1	3	7	YES	YES	Decrease	Recovery	Yedi Pu et al
P8	ALL	CD19	4	1	3	9	YES	YES	Decrease	Recovery	Yedi Pu et al
P9	ALL	CD19	3	0	1	9	YES	YES	Increase	DEATH	Yedi Pu et al
P10	ALL	CD19	3	0	1	8	YES	YES	Decrease	Recovery	Yedi Pu et al
P11	ALL	CD19	3	0	2	7	YES	YES	Decrease	Recovery	Yedi Pu et al
P12	ALL	CD19	3	3	3	6	YES	YES	Decrease	Recovery	Xia Xiao et al
P13	NHL	CD19	3	0	1	8	YES	YES	Decrease	Recovery	Yedi Pu et al
P14	NHL	CD19	3	1	1	7	YES	YES	Increase	Recovery	Yedi Pu et al
P15	NHL	CD19	4	0	1	6	YES	YES	Decrease	Recovery	Yedi Pu et al
P16	NHL	CD19	3	0	1	9	YES	YES	Decrease	Recovery	Yedi Pu et al
P17	NHL	CD19/CD20	4	0	3	3	NO	NO	Decrease	Recovery	Zhang et al
P18	MM	BCMA	4	0	3	10	YES	YES	Decrease	Recovery	Yedi Pu et al
P19	MM	BCMA	3	0	2	5	YES	YES	Decrease	Recovery	Yedi Pu et al

### Management of severe CRS and the role of PE

In the reviewed cases, PE was used as a secondary therapeutic option for patients who did not respond effectively to first-line treatments. Most patients were diagnosed with grade 3 or grade 4 CRS, and 9 patients developed neurotoxicity. All of them were treated with tocilizumab and corticosteroids before PE. The PE procedure involved the removal of plasma with subsequent replacement using fresh frozen plasma or albumin. PE was generally administered over a series of consecutive days, ranging from 1 to 4 times depending on the severity of CRS and the individual response. For instance, Xiao et al. reported that a single patient underwent PE for 3 consecutive days, which resulted in marked improvement in CRS symptoms and a reduction in cytokine levels (17)​.

### Cytokine and clinical outcomes

Before PE treatment, patients exhibited elevated levels of inflammatory markers, particularly IL-6, TNF-α, and IL-10. After undergoing PE, there was a consistent decrease in these cytokines, which correlated with clinical improvements​. In the retrospective cohort study by Pu et al, cytokine levels such as IL-6 and CRP dropped significantly post-PE, indicating the efficacy of PE in alleviating CRS-related symptoms ​ (16). However, it still existed that several patients experienced the CRS progression and eventually died after PE. As shown in the table, the phenomenon that IL-6 levels remained increasing after PE was observed in all those 3 dead patients. This might be explained by the fact that the rate of cytokine removal by PE has fallen below the rate of cytokine production. Therefore, we propose that early intervention of PE might be an effective approach to treat these extremely severe CRS, this opinion will be discussed in the Discussion section.

Although preexisting studies predominantly focus on ALL, the efficacy of PE in treating CAR-T related CRS in NHL and MM was also definite. As shown in **Table 2**, the reduction in IL-6 levels following PE treatment in four NHL patients (average 80%) was comparable to that observed in ALL patients (average 75%), while the future studies might need to expand the sample size of non-ALL cases to further validate the generalizability of PE.

## Review and discussion

In this study, we reported 3 patients with severe CRS after CAR-T cell therapy were treated with PE. Pre-PE, all patients presented with severe CRS. PE was initiated due to failure of conventional treatments like tocilizumab and corticosteroids, which was consistent with literature guidelines.

Post-PE, all 3 patients showed significant improvement, including recovery from neurotoxicity and decreased IL-6 levels. These outcomes aligned with literature reports, where PE effectively removed cytokines and improved clinical outcomes. However, fewer PE sessions were conducted in our cases compared to other studies. Besides, several patients developed adverse outcomes and finally died although PE was administrated. This might be caused by the excessive inflammatory responses, and the rate of cytokine removal by PE was fallen below the rate of cytokine production. This highlighted the necessary of early intervention of PE to treat severe CRS.

### Principles, development, and applications of PE

PE is a blood purification therapy that involves removing the plasma of a patient and replacing it with fresh frozen plasma or other substitute fluids (22). The main objective of PE is to eliminate excessive cytokines, immune complexes, and other harmful substances that contribute to disease progression (23, 24). The use of PE in medicine could date back several decades, initially being used for autoimmune diseases such as systemic lupus erythematosus and myasthenia gravis (25, 26). Gradually, its application has expanded to treat acute kidney injury, toxicological emergencies and blood disorders (27, 28).

In recent years, with the rise of CAR-T cell therapy, PE has been increasingly applied for the management of severe CRS, particularly in patients with conventional treatments failing to control symptoms (16–18). In CAR-T cell therapy, PE is used to remove inflammatory cytokines, such as IL-6, TNF-α and other molecular cytokines that are released after the interaction between cancer cells and CAR-T cells.

Except for CRS caused by CAR-T cell therapy, PE has also been explored for managing CRS caused by other conditions, including viral infections such as COVID-19 (29). In cases of COVID-19, PE has been shown to reduce cytokine levels and improve clinical outcomes, particularly in patients experiencing severe inflammatory responses (30–32). A key difference between CAR-T-related CRS and COVID-19-induced CRS is the underlying immune activation: CAR-T cell therapy causes a rapid proliferation of engineered T-cells that directly attack cancer cells, triggering an inflammatory response; while COVID-19 related CRS results from the body’s response to viral infection, often leading to pulmonary damage and multi-organ failure (14, 33). This distinction in pathophysiology might influence the timing and volume of PE required. For example, PE should be administrated earlier when treating CAR-T related CRS, compared with infection related CRS, as CAR-T cells can exert their cytotoxic effects on tumor cells rapidly. Especially in treating hematologic malignancies, CAR-T cells can encounter and attack tumor cells immediately after being infused into peripheral blood, leading to the release of a large number of cytokines in the short time.

### Other blood purification techniques: focus on HF and HDF

In addition to PE, other blood purification techniques like hemofiltration (HF) and hemodiafiltration (HDF) have been used for managing CRS. HF operates using convection to remove small molecules and excess fluid from the bloodstream, which is particularly useful for fluid overload or the removal of small molecular toxins, such as some inflammatory cytokines (34–36). However, HF is less effective in clearing large molecular weight cytokines, which limits its utility in the treatment of severe CRS (37). On the other hand, HDF combines the mechanisms of both diffusion and convection, which allows for more comprehensive clearance of small and medium-sized molecular weight cytokines (38, 39). HDF is often favored when a more extensive removal of inflammatory cytokines is needed. However, HDF requires more complex equipment and procedures, increasing its resource demands and operational challenges.

### Advantages and disadvantages of PE compared to HF and HDF

Each blood purification technique has its unique advantages and disadvantages in the management of CRS. PE is particularly advantageous for rapidly removing large molecular weight cytokines associated with severe CRS, especially in those patients who fail to respond to conventional therapies. This makes PE highly effective in improving symptoms such as fever, hypoxia and coagulopathy. Also, PE might be a feasible method to avoid or alleviate neurotoxicity, as it can directly reduce the damage to the blood-brain barrier caused by inflammatory cytokines. However, PE also has several drawbacks. Firstly, it is not effective in removing small molecule toxins and can be technically challenging, as it might require specialized equipment and multiple sessions of plasma replacement. Additionally, PE carries a risk of hemodynamic instability, which requires close monitoring during the procedure. Compared with PE, although HF is simpler and easier to perform, it is limited in its ability to clear large molecular cytokines (38). It is effective in addressing fluid overload and removing small molecular toxins, but its role in CRS treatment is less prominent. HDF combines the progress of both diffusion and convection, offering a more comprehensive solution for removing both small and medium molecular cytokines, making it a strong option for severe CRS. However, it is highly resource-intensive and requires high-end equipment, which might lead to severe complications such as hypotension and bleeding (40). As shown in **Table 3**, we compare the advantages and disadvantages of these techniques, emphasizing their treatment mechanisms, efficacy, patient suitability and operational difficulties​.

**Table 3:** Comparing the characteristics of PE, HF and HDF treating CRS.

Feature	Plasma Exchange (PE)	Hemofiltration (HF)	Hemodiafiltration (HDF)
Treatment Mechanism	Removes cytokines and immune complexes from plasma	Uses convection to remove small molecules and fluid, clearing inflammatory cytokines	Combines diffusion and convection to clear small and medium molecular weight solutes and inflammatory cytokines
Clearing Target	Large molecular weight cytokines	Small molecular toxins and fluid, partial cytokine clearance	Small and medium-sized molecular weight inflammatory cytokines
Indications	Used for severe CRS, especially when drug treatments are ineffective	Effective in acute kidney injury and fluid overload; partial cytokine clearance	Used for severe CRS, particularly when more medium molecular weight toxins need to be cleared
Advantages	Rapid cytokine removal, especially effective when conventional treatments fail; Improve coagulation function	Effective for small molecule toxins and fluid overload, suitable for critically ill patients	Comprehensive removal of both small and medium molecular toxins, adaptable treatment for severe CRS
Disadvantages	Poor small molecule clearance, complex procedure, may cause hemodynamic instability	Limited effectiveness in clearing large molecular cytokines, cannot fully control CRS	Complex procedure, high equipment demand, potential for hemodynamic issues like hypotension
Effect on Neurotoxicity	Effective in clearing cytokines related to neurotoxicity	Less effective on neurotoxicity, mainly removes small molecules	Moderate effectiveness in clearing cytokines related to neurotoxicity, suitable for severe neurotoxic CRS patients
Hemodynamic Impact	May cause hypotension and other hemodynamic instability	Minimal hemodynamic impact, suitable for unstable patients	Greater hemodynamic impact, may require strict monitoring and support
Risks and Complications	Infection, allergic reactions, hypotension, electrolyte imbalances	Limited large molecule toxin clearance, may require combination with other therapies	Complex, may cause hypotension, bleeding, and other complications
Resource Requirements	Requires large amounts of replacement fluids (e.g., fresh frozen plasma) and specialized equipment	Lower equipment requirements, suitable for intensive care	High equipment demand, costly, requires continuous therapy
Reference number	(22–28)	(34–36)	(38–40)

To summarize, although PE is an effective method to remove various cytokines, the accessibility and cost of PE may limit its use due to the need for specialized equipment and expertise. In contrast, HF is less costly and simpler, but less effective in clearing large cytokines. HDF can balance both small and medium molecular toxin clearance, but it also requires complex equipment and may cause hemodynamic instability.

### Discussion on timing, plasma volume replacement and future directions in PE for CAR-T-related CRS

The timing of PE and the volume of plasma replacement are critical factors influencing the success of the treatment in CRS management. Nowadays, PE is most used when conventional therapies fail to control severe CRS. However, according to our experience, early intervention with PE might be able to prevent irreversible organ damage and neurotoxicity caused by continuous damage of cytokines. In our opinion, the timepoint to conduct PE is that the level of IL-6 exceeds 1000 pg/ml, or the increase of IL-6 is over 500 pg/ml within 24 hours. Typically, the volume of plasma exchanged is based on the patient’s body weight and hematocrit, with the following formula: PE Volume (L) = 0.065 × Body Weight (kg) × (1 − Hematocrit). However, for patients with severe CRS, additional sessions or a larger plasma volume may be required (41). The future direction of PE lies in optimizing treatment protocols, including determining the best timing, frequency of treatment and the appropriate volume of plasma to replace. Standardizing these protocols will provide more consistent clinical guidelines for the use of PE in CRS treatment. Furthermore, combination therapies involving PE and other immunomodulatory agents may increase the overall effectiveness while reducing the side effects of individual treatments. Advances in other blood purification technologies may lead to the development of more efficient and user-friendly devices, reducing the complexity of the procedure and making it more accessible and safer for severe CRS patients. Additionally, ongoing research into the exact mechanisms of CRS and how blood purification techniques can be combined with CAR-T cell therapy could significantly improve treatment outcomes in the future.

## Conclusion

In conclusion, the management of severe CRS following CAR-T cell therapy presents a significant challenge in clinical practice. While conventional therapies such as corticosteroids and tocilizumab are commonly used, their effectiveness is still limited in severe CRS patients. In this condition, PE has emerged as a promising therapeutic option in these situations, demonstrating a strong ability to rapidly remove inflammatory cytokines and immune complexes. Looking forward, it is extremely necessary to establish standardized protocols for the use of PE in the treatment of CRS. Determining the optimal timepoint, frequency and volume of PE are critical for clinicians or researchers to conduct PE. In this article, we provide a retrospective review of previous usage of PE to treat CRS caused by CAR-T cell therapy, which might help clinicians and researchers to better understand the role and application of PE.
